# Nuclear engineering workforce in the United States

**DOI:** 10.1002/acm2.13808

**Published:** 2022-10-27

**Authors:** Lawrence W. Townsend, Lori Brady, Janice Lindegard, Howard L. Hall, Elizabeth McAndrew‐Benavides, John W. Poston

**Affiliations:** ^1^ Department of Nuclear Engineering University of Tennessee Knoxville Tennessee USA; ^2^ Nuclear Energy Institute Washington District of Columbia USA; ^3^ American Nuclear Society La Grange Park Illinois USA; ^4^ Department of Nuclear Engineering University of Tennessee Knoxville Tennessee USA; ^5^ Electric Power Research Institute Palo Alto California USA; ^6^ Department of Nuclear Engineering Texas A&M University College Station Texas USA

**Keywords:** nuclear engineering, workforce

## 5.1 INTRODUCTION

This section focuses on the nuclear engineering (NE) workforce, as well as those professionals working in its major subfields, including electricity production using nuclear steam supply systems, nuclear fuel cycle, nuclear security and nonproliferation, nuclear reactors for radioisotope production and testing (including medicine), and radiation shielding design. Examples of areas where nuclear engineers are employed include reactor core design and materials research, nuclear fuel cycle research and development, nuclear materials stewardship, and general‐purpose radiation transport code development for NE, health physics, and medical physics applications. In addition to the commercial power industry, employment sectors for nuclear engineers include national laboratories operated by the Department of Energy (DOE), the National Nuclear Security Agency (NNSA), the military, various other government agencies (e.g., Nuclear Regulatory Commission (NRC) and National Aeronautics and Space Administration), and academia. Therefore, the NE workforce is essential to meeting the nation's needs in these and other vital areas.

## 5.2 DEFINITIONS OF THE PROFESSION

Nuclear engineers commonly work in the disciplines of NE, nuclear security, and nuclear criticality safety, as defined later. NE has significant overlap with health physics and medical physics (described in Chapters 2 and 3, respectively) and is also closely allied with mechanical engineering, materials science and engineering, and accelerator physics.

### 5.2.1 Nuclear engineering

Nuclear engineers conduct research and development activities related to the uses of energy produced from nuclear reactions, whether for electricity generation using nuclear power^1^ or in the military sector, where US Navy nuclear ship propulsion involves the development, supervision, and operation of naval nuclear reactor plants. Overall, the activities of nuclear engineers include the development of nuclear power sources for electricity generation and ship propulsion, radioactive materials for medical and industrial applications, weapons for military applications, and applications in nuclear security. Navy nuclear personnel also serve as Naval Reactor Engineers and as instructors at the Naval Nuclear Power School in Goose Creek, South Carolina, and at naval nuclear reactor prototype training facilities in Charleston, South Carolina or Ballston Spa, New York.

### 5.2.2 Nuclear security

Within the scope of nuclear security, nuclear nonproliferation, and nuclear materials stewardship, nuclear engineers develop, test, evaluate, and operate systems designed to protect critical infrastructure and hazardous materials from malicious acts. Detailed analyses of facility operations and technical capabilities of various nuclear systems are used to evaluate potential concerns with compliance in international agreements, such as the NonProliferation Treaty. Military entities and International Atomic Energy Agency (IAEA) personnel are both involved in treaty verification activities and actively conduct inspections to reduce the threat of covert production of nuclear weapons and improvised nuclear devices. Stewardship of nuclear materials includes the use of technical approaches to measure, account for, and maintain safe control of nuclear materials, especially weapons‐usable nuclear materials. Substantial international attention and action in promoting nuclear security resulted from a number of events over the last few decades, notably the collapse of the former Soviet Union (which led to a long‐term commitment on the part of the United States to cooperative threat reduction), the terrorist attacks of 11 September 2001, and the international consensus to improve nuclear security prompted by the Obama administration's leadership in the Nuclear Security Summits. Nuclear policy, strategy, capabilities, and force posture of the United States, for the next 5–10 years, are usually presented in the Nuclear Posture Review report. The current 2022 version has been transmitted to Congress. However, an unclassified version has not yet been released.^2^

### 5.2.3 Nuclear criticality safety

A nuclear criticality accident occurs when operations that involve fissile material undergo an inadvertent, self‐sustaining nuclear chain reaction,^3^ resulting in a sudden, potentially lethal, release of radiation. Nuclear criticality safety engineers seek to prevent such accidents and mitigate their consequences by analyzing normal and credible abnormal conditions in fissile material operations in order to design safe arrangements and procedures.^4^

## 5.3 GENERAL CHARACTERISTICS OF THE WORKFORCE

Studies of workforce status and requirements have been carried out over the past decade for various sectors of NE.^5^, ^6^, ^7^, ^8^ In addition, an opinion piece in *Power Engineering* ^9^ provided an overview of the aging workforce in the US energy industry and its future ramifications. Although the information provided in these reports includes nonnuclear workers, the most telling statistic provided by the US Department of Labor is that half of the utility workforce was expected to retire within the next 5–10 years. Furthermore, the US Government Bureau of Labor Statistics has forecasted an 8% total decrease in NE jobs (∼1500 jobs) for the decade through 2030.^1^

### 5.3.1 Nuclear engineering

According to the US Department of Labor's *Occupational Outlook Handbook*,^1^ ∼17 200 nuclear engineers were employed in 2020. Of these, about 25% were employed in electric power generation, 18% within the federal government, 15% in scientific research and development, 5% in engineering services, 10% in manufacturing, and the remaining 27% in other areas. In 2022, the workforce was 83% male and 17% female. The distribution by race,^10^ in 2019, was 73% White, 7% Asian, 7% Black or African American, 4% Hispanic, 4% Native Hawaiian/Pacific Islander, and 5% other.

#### 5.3.1.1 Power production

In the United States, the nuclear power industry supports ∼100 000 jobs in a wide range of fields, including engineering and skilled trades.^11^ Of these jobs, ∼4200 are nuclear engineers.^12^ The tremendous growth in the nuclear energy sector during the 1970s and 1980s created a stable and qualified workforce, with the large demand for workers often being met by former military personnel, construction workers, and university graduates. However, growth of the industry, including the construction of additional nuclear power plants, was negatively impacted by the reactor accidents at the Three Mile Island plant in Pennsylvania in 1979 and at Chernobyl in the former Soviet Union in 1986, so that employment levels declined in the early 1990s. Although there was a rebound at the turn of the century with new plant builds, fluctuating economic conditions and the Fukushima reactor accident in 2011 have resulted in some plants being shut down prematurely and limited new plant construction. Thus, as of June 2021, there were 93 operating nuclear reactors in the United States, with two new plants under construction. The Nuclear Energy Institute's 2017 Gaps in the Energy Workforce Survey^13^ found that, in 2016, the industry had two workforce peaks, with fewer employees in the mid‐career age group (age 38–47). As the industry has continued to hire a significant number of new employees, as evidenced by the increasing numbers of personnel in the age 23–37 cohorts, there has been a shift in the industry's bimodal age distribution, with an increase in the younger side of the workforce. However, as in all large industries, mid‐career engineers are needed to fill first‐ and second‐level management positions, as well as other critical individual contributor nuclear specialist positions, so this shortfall may create future hardships in the industry.

In recognition of this pending shortfall, over the past decade, the US nuclear energy industry has focused on workforce planning and development efforts, and these efforts have been successful on many fronts. The United States now has enough pipeline programs in place to offset the steady 6%–7% annual attrition rate for most positions (see Section 3.4). However, not all workforce issues have been resolved. For example, there are indications of insufficient numbers of maintenance and radiation protection professionals entering the workforce to offset the anticipated retirements,^13^ although this situation is difficult to gauge given utilities’ reliance on outside suppliers and vendors. Overall, hiring in the industry has been reduced, with many organizations instead absorbing staff from closed units and, in some cases, reducing their headcount through attrition. These issues need to be further studied as new data become available.

### 5.3.2 Nuclear security

Nuclear engineers employed in nuclear security applications in the United States can be found in the civilian nuclear power industry where they work to assure facility compliance with NRC regulations on nuclear security and safeguards at power plants, uranium enrichment plants, and fuel fabrication facilities and provide nuclear waste stewardship pending final disposition. In addition, a large segment of the NE workforce is involved in a wide range of government work, including in military, civilian, and intelligence departments, and Federally Funded Research and Development Centers, such as the DOE national laboratories. Among these are nuclear engineers and related occupations employed by the NNSA, a semiautonomous agency of the DOE. Its major roles include: (1) overseeing the nation's nuclear weapons stockpile; (2) nonproliferation efforts to control and reduce nuclear weapons danger worldwide; (3) responding to nuclear and radiological incidents and accidents worldwide; and (4) providing militarily effective nuclear propulsion plants for the US Navy.

Nuclear engineers are also found in private sector entities engaged in nuclear technology or with nuclear materials, nonprofit entities (e.g., academia and nongovernmental organizations, such as the Nuclear Threat Initiative), as well as in international agencies, such as IAEA, either as direct employees or as US national laboratories experts detailed to the IAEA.

### 5.3.3 Nuclear criticality safety

Nuclear engineers in nuclear criticality safety applications in the United States are employed in the civilian nuclear power industry, where they work to ensure compliance with applicable federal and state regulations regarding safety of nuclear materials during handling, manufacture, and storage of nuclear materials. Their goal is to ensure that inadvertent critical masses of fissile materials cannot be reached during these periods. Employers of these engineers include vendors manufacturing nuclear fuel for commercial and military use, government facilities storing and performing maintenance on nuclear weapons, and facilities that store spent nuclear fuel. Nuclear Criticality safety training is available through the Nuclear Criticality Safety Program (NCSP) at Lawrence Livermore National Laboratory. Information regarding the NCSP mission, vision, and stakeholders is available.^14^ The actual numbers of personnel serving criticality safety roles in the workforce are unavailable and should be assessed as part of a future workforce study.

## 5.4 EDUCATION AND TRAINING PATHWAYS

Most nuclear engineers hold a Bachelor of Science (B.S.) or higher degree in NE. However, many have undergraduate and graduate degrees in other STEM fields. More broadly, formal education requirements for NE personnel vary from high school diplomas to doctoral degrees, depending on the focus of the employer. Reactor plant operators and nuclear vendor personnel generally possess at least a high school diploma, with many possessing an Associate or other undergraduate degree. Those engineers involved in system design for utilities and manufacturers usually possess one or more graduate degrees or significant graduate education. These members of the workforce also receive extensive training specific to their position and workplaces. Personnel involved in basic and applied NE research generally possess at least a Master of Science degree, whereas many researchers, especially those employed in academia and national laboratories, possess doctoral degrees and have received postdoctoral training.

### 5.4.1 Organizations involved in education

The Accreditation Board for Education and Training (ABET) accredits undergraduate programs in NE. Accreditation involves a comprehensive on‐site review that usually occurs every 6 years and is provided for facilities offering associate, bachelor, and master degree levels. In most cases, NE departments and programs are accredited only at the B.S. degree level, as professional licensure generally requires applicants to have graduated from institutions with ABET accreditation; Ph.D. programs are not accredited by ABET.

Numerous scholarship, fellowship, and grant opportunities exist for students interested in pursuing a career in NE.

- The American Nuclear Society (ANS) and its Sections and Divisions offer more than 30 scholarships for students pursuing associate through Ph.D. degrees. For the academic year 2022–2023, 87 scholarships and fellowships, totaling over $240 000, were awarded.^15^
- The United States DOE, Office of Nuclear Energy, offers scholarships for students pursuing associate and bachelor's degrees and fellowships for graduate students through its University Nuclear Leadership Program (UNLP).^16^ In 2022, UNLP awarded over $5 million for 59 scholarships to undergraduate students attending universities having NE degree programs, 1 scholarship for a student attending an undergraduate 2‐year program, and 28 3‐year fellowships for students pursuing graduate degrees in NE. Moreover, in 2022, Infrastructure Grants totaling over $5.2 million were awarded to 20 university‐led projects for research reactor and infrastructure improvements. In addition, seven university‐led Integrated Research Projects were awarded nearly $25 million to address highly complex technical issues relevant to the DOE Office of Nuclear Energy mission. The U. S. National Laboratories also provide nuclear science and technology competitive programs that provide students with on‐site internships.
- The South Carolina Universities Research and Education Foundation manages the Nuclear Nonproliferation International Safeguards Graduate Fellowship Program (NNIS) and the Rickover Fellowship Program in NE for the NNSA and Naval Reactors Division of DOE.^17^
- Other resources provided for education at the university level include the NNSA academic nuclear security consortia ($5 million per year)^18^ and the NRC, which awarded $9 million in academic grants to support nuclear science and engineering.^19^

### 5.4.2 Undergraduate education

Undergraduate education in NE takes place in a variety of public and private universities and colleges, as well as at some 2‐year institutions. In the 2020 *Nuclear Science and Engineering Education Sourcebook*,^20^ there were 33 NE departments and programs in the United States (10 offer only graduate degrees), of which 23 were accredited by the ABET. A typical B.S. NE major curriculum consists of courses in physics (including nuclear physics), chemistry, mathematics through differential equations, engineering statics and dynamics, reactor physics and systems, thermodynamics and fluid flow, electrical and electronic circuits, radiation protection and shielding, Monte Carlo methods, and computer programing. Laboratory courses typically involve experiments and practical exercises in these areas, many involving hands‐on experience with nuclear reactors, where available.

Data for 2019 gathered by the Oak Ridge Institute for Science and Education (ORISE)^21^ indicated that 622 B.S. degrees were awarded by 24 NE departments and programs. Of these, 569 were from institutions having an NE major, and 57 were from institutions offering an NE option (minor or concentration). This number is consistent with the annual number of degrees awarded since 2012 and is higher than the number awarded in the previous decade. Undergraduate enrollment numbers were ∼1740 in the year 2019, a 3% increase over 2018. These overall numbers suggest that the number of B.S. degrees awarded annually over the next several years should continue to remain above 600.

### 5.4.3 Graduate education

In 2016, 355 M.S. and 161 Ph.D. degrees were awarded by 32 departments and programs, most housed in a limited number of major research universities. Of the 355 M.S graduates, data were reported to ORISE for 305 recipients (86%): foreign nationals comprised 17%, females comprised 15%, and US citizen minorities comprised 13% of the M.S. graduates. At the Ph.D. level, data were reported to ORISE for 158 graduates (98%): foreign nationals comprised 28%, females comprised 16%, and US citizen minorities comprised 16%. At the Ph.D. level, 82% of the US females and 75% of the US male were Caucasian. Only 1 African or Black American male and 15 females (none of whom were African or Black American) were awarded a Ph.D. These figures indicate that females and African or Black Americans were significantly underrepresented in NE at the Ph.D. level. Overall, graduate student enrollment in 2019 was ∼1690 students—numbers comparable to those reported in 2015, but 5% lower than reported in 2018.^21^ These enrollment levels suggested that the number of graduate degrees to be awarded in the near future will remain comparable to recent years. In 2019, 316 M.S. and 194 Ph.D. degrees were awarded. Breakdown of the data by citizenship, gender or race was not available for 2019 in the 2020 report.

### 5.4.4 Postgraduate education

Postdoctoral Research Associate positions, typically 1–3 years in duration, are mainly located in NE academic departments and at national laboratories operated by the US DOE, Department of Defense, NASA, and many other US government agencies. Many of the programs these federal agencies have are managed by ORISE. Details of the numerous offerings available and how to apply are presented on the ORISE website.^22^

### 5.4.5 Alternate pathways

#### 5.4.5.1 Community colleges

In 2007, the United States only had five community colleges with nuclear technology programs. Since that time, the industry has reconstituted this pipeline, and the Nuclear Uniform Curriculum Program (NUCP) now has 32 academic partners.^23^ The NUCP has become the flagship pipeline program for the industry, and its goal is to create a pipeline of entry‐level workers who have received a common level of education and are transferable to any industry member. The program is fully aligned with utility training programs and is sized to graduate an appropriate number of certificated students, enabling utilities to hire cohorts of NUCP graduates into initial training. Some may continue further studies to earn B.S. degrees in NE or other related STEM fields.

#### 5.4.5.2 Military training and education programs

Many NE personnel are trained within the US Navy nuclear propulsion program. Enlisted personnel are generally nuclear power plant operators, supervised by naval officers who serve on ships (aircraft carriers and submarines) deployed around the world. Training generally consists of 6 months of classroom training and an additional 6 months of training and watch‐keeping qualifications on nuclear reactor plant prototypes. Military veterans form one of the traditional pipelines into the nuclear energy industry, as well as into the broader energy industry. In 2008, the Nuclear Energy Institute (NEI) undertook a first‐of‐a‐kind effort to develop an Agreement of Understanding with the US Navy to streamline identification of Navy personnel for employment in the industry. The agreement was signed in 2012, and monthly emails now are sent to nuclear utilities with the contact information of officers wishing to work in nuclear energy. Concurrently, the Center for Energy Workforce Development developed the Troops to Energy program. This program developed a career website that provides all industry job postings and a database for industry to search through interested veterans’ resumés. It also has a translator that aligns military occupation codes to energy career equivalents. Counseling is available for interested veterans who need help developing a resume or understanding how their skills translate to industry job titles. It is anticipated that the Troops to Energy recruitment effort and Veterans in Energy, an organization developed to provide transition, retention, and professional development support to military veterans who have chosen energy careers, will support one another.

### 5.4.6 Certifications and licensure

Within the United States, licensure of nuclear engineers is carried out by state professional licensing boards. These boards confer the Professional Engineer (P.E.) license in NE based upon a combination of requirements in education, experience, and exams that are often unique to a particular state.^24^ For example, in Tennessee, an applicant for a P.E. license must have graduated from an ABET‐accredited, or equivalent, 4‐year engineering program. In neighboring Virginia, an applicant can apply if they have graduated from a “related science curriculum” and have the required minimum qualifying engineering experience. Continuing education requirements, which vary by state, exist for retaining licensure. Nonetheless, all personnel who operate or supervise the operation of the controls of a commercial, test, or research reactor in the United States, except for test reactors controlled by the DOE, must be licensed to do so by the NRC. As of July 2019, there were ∼3900 NRC‐licensed reactor operators in the United States.^25^

### 5.4.7 Continuing education

Continuing education is generally not required in NE, except for maintaining professional licensure. However, a variety of technical courses are available from DOE national laboratories, professional organizations, and university NE departments and programs.

## 5.5 PROFESSIONAL ASPECTS OF RELEVANCE TO WORKFORCE SUPPLY

### 5.5.1 Professional organizations

Nuclear engineers in the United States typically have membership, and participate, in the activities of one or more organizations related to the profession. These include, but are not limited to, the ANS, the Institute of Nuclear Materials Management, the American Society of Mechanical Engineers, the Society for Maintenance and Reliability Professionals, the American Society for Engineering Education, and the Health Physics Society.

### 5.5.2 Interdependencies with other radiation professions

Although many in the NE profession have one or more degrees in NE, there are many who are academically trained in other STEM fields, especially physics, applied physics, and other engineering disciplines. These interdependencies enrich the profession by giving broader exposure to aspects of NE applications in a variety of areas other than power production to students. Academic training in health physics/radiological engineering, medical physics, and medical imaging is also provided by some NE departments or programs (Georgia Institute of Technology, Idaho State University, Oregon State University, Texas A&M University, University of Illinois, University of Massachusetts Lowell, University of Missouri Columbia, and the University of Tennessee). NE faculties often include nuclear physicists (theoretical and experimental), applied physicists, electrical engineers, mechanical engineers, chemists, and even applied mathematicians. These faculty members train students in the use of radiation transport codes for medical applications and designs of accelerator‐based treatment facilities, the operation and use of imaging systems for medical diagnostics, development of detection systems for measuring radiation levels, and methods of assessing risk from radiation exposures on Earth and in space.

## 5.6 CURRENT STATUS AND FUTURE OUTLOOK

The bimodal age distribution described in Section 5.3.1.1 points out the potential lack of experienced engineers to fill the mid‐level positions as older personnel retire, especially within the nuclear utility sector. Overall, the US Department of Labor projects a decrease of 8% in the number of NE jobs over the next decade. Current production of nuclear engineers appears sufficient to meet the nation's future needs unless the demand for nuclear power production increases and requires the addition of new, additional nuclear power plants.

## 5.7 SUMMARY AND RECOMMENDATIONS

NE has matured as a discipline and has largely recovered from the downturn in professional numbers seen after the accidents at Three Mile Island and Chernobyl nearly four decades ago. The hiring of young engineers into the existing workforce appears to be adequate for the current demand; however, a gap in mid‐level management may appear due to the large numbers of senior personnel who are expected to retire over the next decade. In the near term, a reduction in personnel due to plant closures is somewhat offset by shifting workforce roles from operators to personnel involved in decommissioning these plants. In the long term, fewer plants means that fewer employees are needed to operate and maintain them, resulting in decreasing job numbers. However, as there have been no comprehensive studies of the workforce in nearly a decade, clearly these contradictory issues need to be addressed.

The recommendations in the following are consensus expert opinions on actions needed to ensure that the NE profession will be able to meet the nation's future needs. The authors intentionally declined to recommend detailed methods, timelines, responsibilities of individual organizations, and funding sources. These complex subjects are outside the scope of this Review and, indeed, the authors were prohibited from activities that could be construed as advocacy.

**The authors recommend the following items to ensure the future adequacy of the nation's professional NE workforce**.

1. The profession should carefully monitor for a potential lack of experienced engineers to fill the mid‐level positions as older personnel retire, especially within the nuclear utility employment sector. Although, currently, there appears to be an adequate supply of entry‐level workers, the sector may need to implement professional development activities to ensure a sufficient cohort of adequately prepared mid‐level engineers.
2. Employers should continue to take steps to preserve their corporate knowledge and experience bases as the wave of retirements of older workers continues.
3. Sufficient support should be available to maintain an appropriate capacity of academic NE programs. Although student enrollment in NE departments and programs is currently robust, a contraction of the NE job market could jeopardize academic NE programs, for example, following the shuttering of additional nuclear plants in the United States in the future. Reduced employment opportunities for graduating students would eventually reduce the numbers of students in the training pipeline and likely result in a reduction in the number of remaining NE degree programs, as occurred in the 1980s and 1990s.
4. As with several other radiation professions, NE needs to become more inclusive and diverse, especially with respect to the numbers of females and minorities. This includes increasing the diversity of the education pipeline, which is lacking both in faculty representation and student populations.

## AUTHOR CONTRIBUTION

All the authors listed have contributed directly to the intellectual content of the manuscript.

## CONFLICT OF INTEREST

No conflict of interest.

## Data Availability

Data sharing not applicable—no new data generated.

## References

[1] U.S. Bureau of Labor Statistics . Occupational Outlook Handbook. U.S. Bureau of Labor Statistics. 2022. Accessed July 26, 2022. https://www.bls.gov/ooh/architecture‐and‐engineering/nuclear‐engineers.htm

[2] U.S. Department of Defense . Fact Sheet: 2022 Nuclear Posture Review and Missile Defense Review. U.S. Department of Defense. 2022. Accessed July 22, 2022. https://media.defense.gov/2022/Mar/29/2002965339/‐1/‐1/1/FACT‐SHEET‐2022‐NUCLEAR‐POSTURE‐REVIEW‐AND‐MISSILE‐DEFENSE‐REVIEW.PDF

[3] Knief RA . Nuclear Criticality Safety: Theory and Practice. American Nuclear Society; 1985.

[4] Wikipedia Contributors . Nuclear Criticality Safety. Wikipedia. The Free Encyclopedia. April 2, 2022, 10:26 UTC. 2022. Available at: Accessed July 26, 2022. https://en.wikipedia.org/w/index.php?title=Nuclear_criticality_safety&oldid=1080619686

[5] American Physical Society . Readiness of the U.S. Nuclear Workforce for 21st Century Challenges. American Physical Society. 2008. Accessed May 5, 2022. https://www.aps.org/policy/reports/popa‐reports/upload/Nuclear‐Readiness‐Report‐FINAL‐2.pdf

[6] McAndrew‐Benavides E . Nuclear Energy Institute. NEI's 2015 Nuclear Workforce Survey Presentation to the NRC. Nuclear Energy Institute; 2015. Accessed January 15, 2019.

[7] Council NR . Assuring a Future US‐Based Nuclear and Radiochemistry Expertise. National Academies Press; 2012.

[8] Weber MF . Meeting regulatory needs. Health Phys. 2017;112(2):176‐181. 28027158 10.1097/HP.0000000000000620

[9] Ray R . Who will replace nuclear power's aging workforce? Power Eng. 2015;8(1). Accessed October 25, 2022. https://power-eng.com/nuclear/who-will-replace-nuclear-power-s-aging-work-force/

[10] Career Explorer by Sokanu . Nuclear Engineer Demographics in the United States. Career Explorer by Sokanu; 2022. Accessed July 26, 2022. https://www.careerexplorer.com/careers/nuclear‐engineer/demographics/

[11] Nuclear Energy Institute . Creating American Jobs *–* Nuclear Energy Institute. Nuclear Energy Institute; 2017.

[12] U.S. Bureau of Labor Statistics . Occupational Employment and Wages. U.S. Bureau of Labor Statistics; 2021. Accessed July 26, 2022. https://www.bls.gov/oes/current/oes172161.htm

[13] Nuclear Energy Institute . 2017 Gaps in the Energy Workforce Survey. Nuclear Energy Institute; 2017.

[14] U.S. Department of Energy . The Mission and Vision of the United States Department of Energy Nuclear Criticality Safety Program. FY 2019–2028. U.S. Department of Energy. 2019. Accessed July 22, 2022) https://ncsp.llnl.gov/sites/ncsp/files/2021‐04/ncsp_mission_vision.pdf

[15] American Nuclear Society . 2022–2023 ANS Scholarship Recipients. American Nuclear Society. 2022. Accessed 20 July 2022). https://www.ans.org/scholarships/recipients/

[16] U.S. Department of Energy . Nuclear Energy University Program. U.S. Department of Energy. 2022. Accessed July 22, 2022. https://neup.inl.gov/SitePages/Home.aspx

[17] The South Carolina Universities Research and Education Foundation (SCUREF) . SCUREF Programs . The South Carolina Universities Research and Education Foundation (SCUREF). Accessed July 22, 2022. https://www.scuref.org/scuref‐programs/

[18] National Nuclear Security Agency (NNSA). *NNSA Renews University Consortium Grant Research and Development into Nuclear Science, Engineering, and Security*. National Nuclear Security Agency (NNSA). Accessed July 23, 2022. https://www.energy.gov/nnsa/articles/nnsa‐renews‐university‐consortium‐grant‐research‐and‐development‐nuclear‐science

[19] U.S. Nuclear Regulatory Commission . NRC Library Document Collections 2022. U.S. Nuclear Regulatory Commission. 2022. Accessed July 22, 2022). https://www.nrc.gov/reading‐rm/doc‐collections/news/2022/index.html#144656

[20] American Nuclear Society and U.S. Department of Energy . 2020 Nuclear Science and Engineering Education Sourcebook. American Nuclear Society and U.S. Department of Energy. 2020. Accessed July 22, 2022. https://neup.inl.gov/SiteAssets/FY2020_Documents/Nuclear.S%26E.Education.Sourcebook.2020.pdf

[21] Oak Ridge Institute for Science and Education . Nuclear Engineering Enrollments and Degrees Survey, 2019 Data, NE Brief #82, September 2020. Oak Ridge Institute for Science and Education. 2020. Accessed July 2024. https://orise.orau.gov/stem/reports/ne‐brief‐82‐2019‐data.pdf

[22] Oak Ridge Institute for Science and Education . STEM Internships and Fellowships. Oak Ridge Institute for Science and Education. 2022. Accessed July 23, 2022. https://orise.orau.gov/internships‐fellowships/index.html

[23] Nuclear Energy Institute . Schools in the Nuclear Uniform Curriculum Program. Nuclear Energy Institute. 2015. Accessed July 26.2022. https://www.nei.org/CorporateSite/media/filefolder/nucp/nucp‐schools.pdf?ext=.pdf

[24] National Council of Examiners for Engineering and Surveying . Engineering Licensure. National Council of Examiners for Engineering and Surveying. 2022. Accessed Jul 26, 2022. https://ncees.org/engineering

[25] U.S. Nuclear Regulatory Commission . Operator Licensing. U.S. Nuclear Regulatory Commission. 2022. Accessed July 26, 2022. https://www.nrc.gov/reactors/operator‐licensing.html

